# Can non-physician advanced retrieval practitioners (ARP) acquire and interpret diagnostic views of the lungs with sufficient quality to aid in the diagnosis of pneumothorax in the pre-hospital and retrieval environment?

**DOI:** 10.1186/s13049-020-00797-8

**Published:** 2020-10-16

**Authors:** James Ronaldson, Christopher E. J. Moultrie, Alasdair R. Corfield, Evelyn McElhinney

**Affiliations:** 1 ScotSTAR, 180 Abbotsinch Road, Glasgow, PA2 3RY USA; 2 Emergency Department, University Hospital Wishaw, Wishaw, UK; 3 Emergency Department, Royal Alexandra Hopsital, Paisley, UK; 4 Department of Nursing & Community Health, Glasgow Caledonian University, Glasgow, UK

**Keywords:** Emergency medical services, Pneumothorax, Diagnostic imaging

## Abstract

**Background:**

As an adjunct to physical examination, ultrasound is a potentially attractive option for diagnosing pneumothoraces in the pre-hospital and retrieval environment – and could confer a benefit to patient safety. However, the published evidence supporting non-physicians use of ultrasound in this setting is limited.

**Aim:**

We aimed to establish if Advanced Retrieval Practitioners (non-physicians) could acquire ultrasound views of the lungs and interpret them with sufficient quality to diagnose pneumothorax in the pre-hospital and retrieval environment when compared to expert review.

**Method:**

The study consisted of an observational trial from April 2017 to April 2018. Twelve (12) patients bilateral lung ultrasound images (24 images) were randomly selected from 87 patients assessed using Point of Care Ultrasound (POCUS) by three Advanced Retrieval Practitioners in the Pre-hospital and Retrieval environment. Two expert reviewers’ evaluated these images to determine ARPs ability to acquire diagnostic quality images and interpret them correctly. CXR results of patients in whom lung ultrasound was undertaken were recorded as the reference standard investigation.

**Results:**

Within the 22 images considered adequate by the Advanced Retrieval Practitioners, 19 (86.4%, one-tailed McNemar test *p* = 0.125) were considered adequate on expert review. Of the 19 images mutually considered as adequate, both the Advanced Retrieval Practitioners and the reviewers identified two pneumothoraces which were subsequently confirmed on chest x-ray (Sensitivity 100% and Specificity 100% in technically adequate images). One pneumothorax was detected on CXR in a patient with inadequate ultrasound images. Advanced Retrieval Practitioners were therefore able to both obtain adequate images and correctly diagnose pneumothorax in the pre-hospital environment with 66.6% sensitivity (95%CI 66.6–100%) and 100% specificity (95%CI 81.0–100%) compared to expert review.

**Conclusion:**

Advanced Retrieval Practitioners (non-physicians) can obtain diagnostic views of the lungs of sufficient quality to diagnose the presence, or particularly the absence, of pneumothorax in the pre-hospital and retrieval environment. Although Advanced Retrieval Practitioners were less accurate than the expert reviewers at interpreting the quality of the ultrasound images, the result was not statistically significant, despite the ARPs possibly having been at a methodological disadvantage.

## Background

Diagnosis of pneumothoraces based on clinical assessment alone has limited value, particularly in the detection of small pneumothoraces [1]. The addition of ultrasound assessment to increase the sensitivity of pneumothorax detection has become established practice within the hospital environment [2]. In the pre-hospital setting, using clinical assessment to diagnose pneumothoraces incurs additional challenges mainly related to the environment. Failure to identify pneumothoraces in pre-hospital care has the potential for more significant consequences than the in-hospital environment, due to the potential for worsening of the pneumothorax during transfer. Air transfer is considered to present a particular risk due to changes in atmospheric pressure associated with flight. Despite the diagnostic challenge, and perhaps related to clinician awareness of the implications of missed diagnosis, tension pneumothorax is often over-diagnosed and over-treated in the pre-hospital environment [3]. This has the potential for patient harm, particularly pertaining to the infection risk from emergency surgical procedures performed pre-hospital. Point of Care Ultrasound (POCUS) may facilitate more accurate diagnosis of pneumothorax when compared to clinical examination: potentially reducing unnecessary interventions and their associated risk of morbidity [4].

O’Dochartaigh’s [5] review highlighted POCUS training to be feasible for both physicians and non-physicians. Two small observational studies conducted in clinical environments have demonstrated that nurses and paramedics are able to acquire adequate images and interpret them as accurately as those performed by physicians [6, 7]. Within the United Kingdom (UK) there is limited published evidence supporting non-physicians clinical diagnostic use of ultrasound in the pre-hospital and retrieval environments. Studies conducted have been set in controlled, simulated conditions on either cadavers or healthy models [8].

Advanced Retrieval Practitioners form an integral part of the Emergency Medical Retrieval Service (EMRS) clinical team - delivering advanced resuscitation, stabilisation and transfer of critically ill patients. The EMRS forms part of the national critical care transfer service in Scotland – provided by the Scottish Specialist Transfer and Retrieval (ScotSTAR) division of the Scottish Ambulance Service. The EMRS has two main clinical roles:

- The principal purpose of the service is the support of rural hospitals and General Practitioners through delivery of a critical care advice and retrieval service (secondary retrieval). Transport for secondary retrievals is provided via helicopter, aeroplane and road vehicles. This patient group contains a mix of major trauma and multi-organ failure medical patients.
- Additionally, the service operates a pre-hospital critical care and transfer (primary retrieval) service, predominantly for major trauma patients. This service includes the delivery of pre-hospital anaesthesia, blood and resuscitative surgical procedures.

In the EMRS context, an Advanced Retrieval Practitioner (ARP) is a nurse or paramedic who has gained a substantial amount of experience working in primary and secondary retrieval; including critical care aeromedical transfers. Routinely they will work as part of a Consultant led team but can also work autonomously within their scope of practice when required.

In 2010, Brooke et al. [8] conducted a narrative review of the literature to identify clinical studies that examined the use of ultrasound by non-physicians in the pre-hospital environment. They concluded that paramedics from outside the UK are able to perform POCUS with images of sufficient quality to positively identify lung pathology found in critically ill patients. Lyon et al’s study [9] demonstrated that all members of a critical care transport team could acquire and retain the skills necessary for using ultrasound to detect the sliding lung sign (SLS) - a sensitive indicator which occurs due to movement of the visceral pleura directly on the parietal pleura [2] - the presence of which excludes pneumothorax.

In the pre-hospital and retrieval environment clinicians must additionally manage the challenges presented by inclement weather, cognitive distractions and limited space, amongst others. Roline et al’s [10] study suggested that significant limitations to the application of POCUS for detecting pneumothorax in their Helicopter Emergency Medical Service (HEMS) was lack of time and aircraft space. Lyon et al. [9] stated that it would be prudent to evaluate the effects of vibration, motion and visibility during active transport as they may impact the accuracy of the SLS for aiding detection of pneumothorax.

Previous studies [11–13] reviewing non-physicians use of lung US have been conducted in specifically controlled research environments. They all used pre-recorded lung imaging videos. Chin et al. [14] used healthy simulated patients, while Lyon et al. [9] used cadaveric models. The generalisability of these studies to the real-world clinical pre-hospital environment is limited, and so the potential decrease in sensitivity, specificity, and accuracy in diagnosing pneumothorax under the challenges of the pre-hospital environment remaining unknown.

In this study, we aimed to assess the feasibility of non-physicians working within a UK pre-hospital service to undertake pre-hospital ultrasound pneumothorax diagnosis in a live clinical environment, and assess the accuracy of the pneumothorax diagnosis.

## Methodology

We conducted an observational study in which the ultrasound images and their associated interpretations generated by a team of three ARPs during the course of their normal clinical practice were retrospectively analysed by two expert reviewers to determine their technical quality and diagnostic accuracy.

### Population

**Fig. 1:**
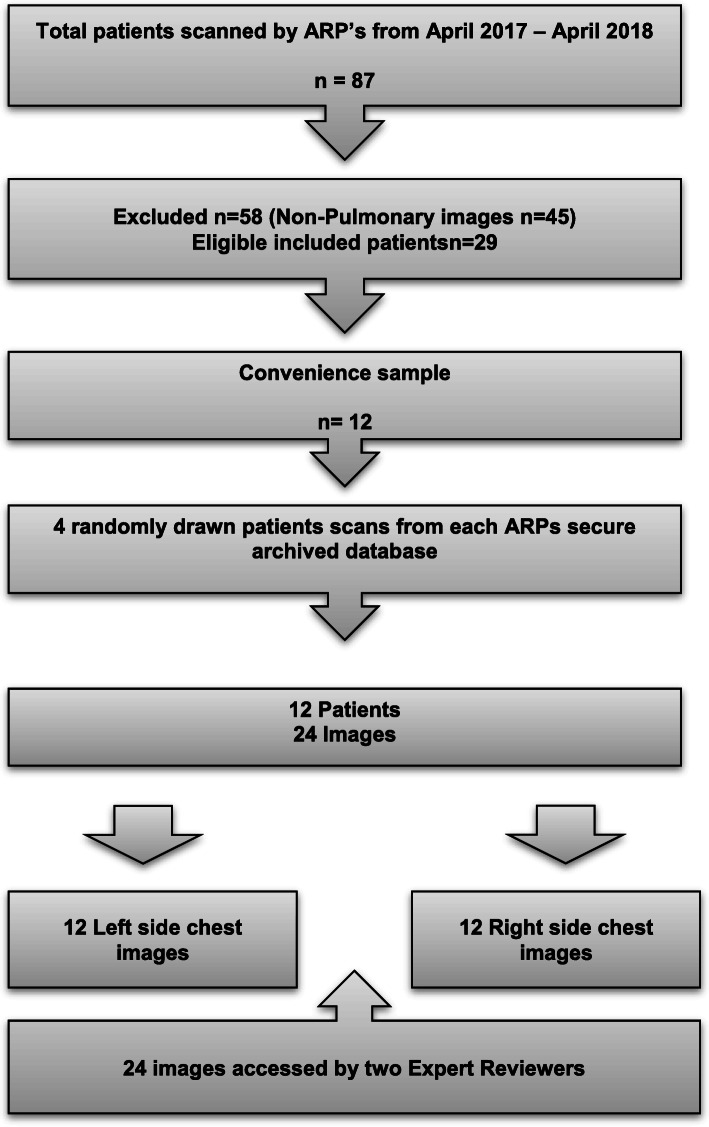
Consort diagram of sampling strategy

Ultrasound images captured by three ARPs during a 12-month period from April 2017 to April 2018. The time available for expert review was limited by the ongoing clinical duties of the reviewers. Review of 12 patients (24 images) was considered achievable within this constraint and so 12 patients were selected as a convenience sample. To reduce selection bias in this relatively small sample and to stratify images between ARPs from different clinical backgrounds: four patients from each ARP’s obtained ultrasound videos (12 patients total) were selected by random draw, performed by a blinded staff member. This encompassed a total of eight images (two images from each of the four patients) per ARP (See Fig. 1) to a total of 24 images (left and right lung studies for 12 patients).

### Image acquisition

ARPs, during the course of their clinical work, obtained lung images of patients in whom there was clinical suspicion of pneumothorax. Ultrasound images were obtained using a GE (General Electric company, Boston, USA) V-scan dual-probe, pocket-sized, battery-powered ultrasound machine. The high-frequency linear array transducer was used for each examination, as is standard practice for lung ultrasound to diagnose pneumothorax. As a result of the challenges of the pre-hospital environment, the ARPs performed an abbreviated anterior ultrasound examination of the chest. This recognized practice involves placing the linear probe in the second intercostal space on both the left and right chest with the patient preferably in the supine position [15]. The images were saved in real-time to the device as video clips.

**Table:** 

Inclusion Criteria	
• Adult patients (> 18 years of age) obtained by an Advanced Retrieval Practitioner. • Ultrasound video of lungs obtained and saved to file • Trauma and Medical	
Exclusion Criteria	
• Images from patients < 18 years of age • Patients with saved static image of lungs • No saved video of the lungs available • Abdominal and Cardiac saved images	

Retrospective descriptive data was obtained from the ScotSTAR EMRS electronic patient record database. The specific information pertaining to the conduct, and challenges, of the ultrasound scan were obtained from the ARPs’ ultrasound electronic database.

### Evaluation tool

Permission was kindly granted by Dr. Jacob Quick to use an evaluation tool from their prospective observational trial [6]. The characteristics of the participants and trial setting were closely representative to this project. Quick et al’s study sought to evaluate the ability of prehospital, in-flight thoracic ultrasound to identify pneumothorax when compared to chest X-ray and computed tomography (CT). Physicians and non-physicians completed the tool without any documented concerns. The tool consists of 5 binary yes/no questions in relation to both left and right sides of the chest. It uses a combination of both positive and negative sonographic signs to improve the accuracy of ultrasound-based diagnosis of pneumothorax. The sonographic nomenclature of the signs used in the tool is supported by expert consensus [16]. Prior to review of the images, this study’s expert reviewers agreed that the tool was clear and understandable. To objectively measure the diagnostic quality of the images, the views were denoted as satisfactory or unsatisfactory based on the ability to clearly visualise the pleural line – the same assessment used previously by previous studies [6, 14].

### Review of the images

Each ARP clinically interpreted the images at their initial point of care assessment with the patient. The images and the ARP’s interpretation thereof were recorded prospectively in the electronic database. The ARPs’ interpretations were then immutably transferred to the ultrasound evaluation tool at a later time.

The two expert reviewers independently reviewed the images and separately completed their evaluation tools. One expert reviewer was an EMRS Emergency Medicine Consultant and the other a non-EMRS Emergency Medicine Consultant who is the RCEM regional ultrasound lead. Each reviewer was blinded to both the ARP and the other reviewer’s interpretation of the ultrasound images, and also to the subsequent chest x-ray (CXR) result. Specialist radiologists reported all CXRs, the results of which were obtained as the reference standard for the study.

### Data analysis

Data analysis was undertaken using SPSS version 24 (IBM Corporation, Texas, USA) and the open-source R language and environment for statistical computing (R Foundation for Statistical Computing, Vienna, Austria, 2018). The study design did not permit the ARPs to attain superior diagnostic accuracy to the reviewers so a one-tailed hypothesis test was used. A *p* value of < 0.05 is considered statistically significant. The degree of agreement between the two expert reviewers interpretation of the images is analysed using the Cohen Kappa statistic.

### Ethical approval

The Scottish Specialist Transfer and Retrieval (ScotSTAR) Research and Development group reviewed the projects proposal and approved accessing stored ultrasound images. Glasgow Caledonian University School of Health & Life Sciences Nursing and Community Health ethics committee granted ethical approval for the study. (HLS/NCH/17/025).

## Results

Between April 2017 and April 2018 a total of 87 patients were scanned, of which, 29 were eligible for inclusion into the study. These images had been anonymised and stored in a secure electronic format at the researcher’s operations base. Twelve patients images were included in the analysis (Fig. 1).

Of the twelve patients whose pre-hospital lung ultrasounds were selected for analysis, 17% (*n* = 2) were female and 83% (*n* = 10) were male. Age demonstrated a mean of 46 and mode of 36 years. The National Early Warning Score (NEWS) demonstrated a median of 8.5 with an interquartile range (IQR) of 8.

The indication for scan was 50% trauma (6/12) and 50% medical (6/12). Thoracic trauma was distributed between penetrating (*n* = 2) and blunt (*n* = 4) trauma. Medical indications for lung ultrasound related to circulatory (n = 2) and respiratory compromise (n = 4).

**Fig. 2:**
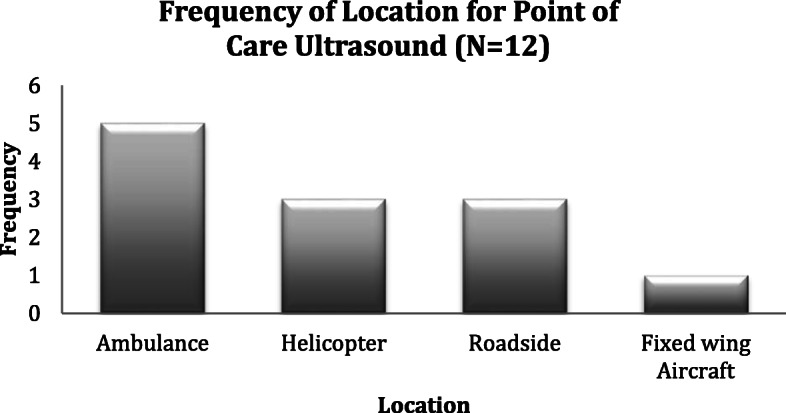
Frequency of Location for Point of Care Ultrasound (*N* = 12)

**Fig. 3:**
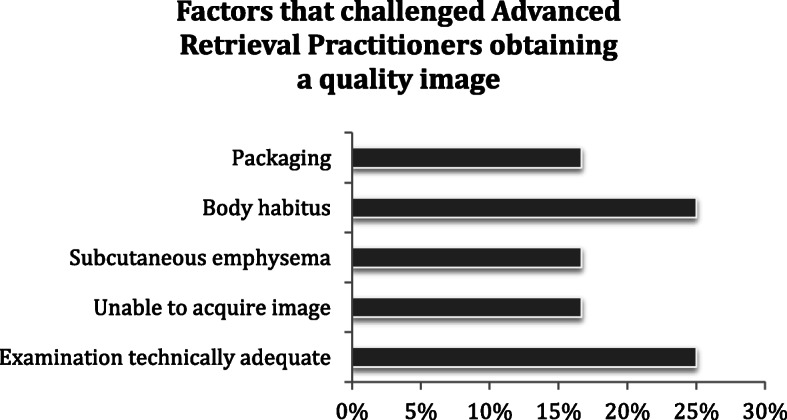
Factors that challenged Advanced Retrieval Practitioners obtaining a quality image

Pertaining to the ultrasound environment: the largest proportion of ultrasound images was acquired inside a land ambulance (41.7% *N* = 5 – See Fig. 2). Three patients (25%) had their images acquired outside at the “roadside” – comprising two urban street locations and a rural industrial location (Fig. 2). Patient packaging challenged ARPs obtaining adequate views in 16.6% of the images taken (Fig. 3). The ARP’s commented on two specific factors that challenged their ability to acquire diagnostic quality images; patients’ body habitus and the presence of subcutaneous emphysema – see Fig. 3).

### Expert reviewer inter-reliability

There was 100% inter-observer agreement (Kappa = 1.00) between the two experts in assessment of image adequacy and accuracy.

### ARP image adequacy

The Advanced Retrieval Practitioners considered twenty-two images obtained to be adequate. Of these, nineteen (86.4%) were also considered diagnostically adequate on expert review. The difference was not statistically significant (McNemar one-tailed *p* = 0.125).

### ARP image accuracy

Within the nineteen images considered mutually adequate, the Advanced Retrieval Practitioners identified two pneumothoraces, which were also identified by both expert review and CXR (Comparative sensitivity 100%).

There were no false positives from the ARPs or expert reviewers (Comparative specificity 100%).

Within patients in whom the pre-hospital ultrasound images were not of diagnostic quality, CXR subsequently demonstrated one pneumothorax. Therefore, when image adequacy was also taken into account, two out of three CXR diagnosed pneumothoraces were identified by the ARP’s using ultrasound (overall sensitivity 66.6%).

This project therefore demonstrated that for this group of patients Advanced Retrieval Practitioners were able to obtain adequate images in the pre-hospital environment *and* correctly detect pneumothorax with:

- 86.4% accuracy when compared to expert review.
- 66.6% sensitivity (95%CI 57.5–100%).
- 100% specificity (95%CI 81.0–100%).

## Discussion

### Summary of main findings

Our data shows that paramedics and nurses have the ability to use POCUS effectively in the pre-hospital and retrieval environment. This effectiveness is dependent on a two-step process. Firstly they are capable of acquiring diagnostic quality images of the lungs. Secondly they are able to interpret images and correctly diagnose the presence or absence of pneumothorax when compared to CXR.

### Strengths and weaknesses

The use of the CXR as a gold standard was a methodological limitation of the study as it is recognized that both ultrasound itself and CT are more sensitive in diagnosing pneumothorax. During review of the data, it was identified that there was one pneumothorax which had been identified on CXR which had been noted by the ARPs - but which, on expert review, had been considered to be an inadequate image. As a result, this was considered a ‘missed’ pneumothorax because, according to the methodology, the expert reviewers were assumed to be always correct; thus, any difference in interpretation would always deem the ARPs to be wrong. This influenced the ARPs’ overall sensitivity of 66.6%. It may have been the case that the ARPs had more information at their disposal, either in the form of clinical information – having been at the scene, with the patient at the time of the scan or had used their interpretation of adequate ultrasound images in the scan beyond that captured for in the video clips – with which to diagnose pneumothorax and which would positively account for the ARPs ability to correctly diagnose pneumothorax from a seemingly inadequate image. Aside from this methodological consideration, the ARP’s diagnostic performance emulated a successful previous “real world” pre-hospital study in relation to their sensitivity and specificity (5-Quick et al. 2016 - Sensitivity 68% (CI 0.46–0.85).

Specificity 96% (CI 0.90–0.98 and Accuracy 91% (CI 0.85–0.95).

A limitation to the study was the restricted sample of available Advanced Retrieval Practitioners that had received training and whom had gained adequate exposure in performing POCUS in the pre-hospital and retrieval environment. This limitation meant that the sample was small and had to be convenient due to the small pool of competent practitioners and limited available time from the expert reviewers. A future study could compare the inter-ARP agreement of images, addressing heterogeneity between the ARP’s training. In this study two practitioners had completed level one ultrasound training days endorsed by the Royal College of Emergency Medicine. One of the practitioners had also successfully completed a log-book and triggered assessments to be deemed competent with equivalence (by appropriate support and supervision at the clinician’s place of work) to Royal College of Emergency Medicine (RCEM) Core Level One ultrasound curriculum. The third practitioner had attended Focused Intensive Care Echo (FICE) training.

### Implications for practice

The scans were conducted on real patients during real clinical practice in the pre-hospital environment for the contemporaneous diagnosis of emerging pathology. Images were acquired outside the hospital on a range of transport platforms including land ambulances, rotary wing and fixed wing, in addition to the outdoor environment itself. This contrasts previous studies which were performed on cadavers, or in controlled environments.

The ARP’s commented that packaging, body habitus and subcutaneous emphysema presented challenges to acquiring images. In the transport platforms used, the patients’ left side is relatively inaccessible due to the proximity of rigid stretcher and vehicle structures. Careful packaging of patients to provide pain relief, clot stability and safety is essential in pre-hospital and retrieval medicine. To avoid interfering with this intervention, clinicians should aim to achieve primary image acquisition prior to patient packaging. “Image access windows” – access points to critical areas of the patient’s anatomy for the purposes of obtaining ultrasound images created through the patient packaging equipment - can then be used to continue dynamic ultrasound monitoring of the patient. Obtaining adequate ultrasound images in obese individuals can be more difficult due to attenuation of the signal over a longer skin-to-organ distance [17]. Similarly, subcutaneous emphysema acts as a barrier of air affecting acoustic impedance; scattering the ultrasound waves and preventing the composition of deeper ultrasound images [18].

### Implications for research

Operator dependence of ultrasound is a well described limitation [19]: it is a ‘real-time’ imaging modality, relying heavily on immediate interpretation of the moving ultrasound image rather than later review of static ‘hard copy’ imaging. Retrospective analysis of saved imaging is a long established and generally effective method of assessing report accuracy for many medical imaging modalities [20]. However, this approach caused difficulties for the expert reviewers who did not have the benefit of real-time images, instead having access to only 3 second “looped” video clips. The study’s choice of expert reviewers may not be considered gold standard, in that they were not radiologists in hospital. However, it was a deliberate, pragmatic choice to use ultrasound trained Emergency Medicine (EM) physicians as the reference standard for patients in the pre-hospital environment, where a radiologist is not available and the immediate, diagnostic interpretation of point-of-care ultrasound is therefore carried out by EM, ICM or anaesthesia physicians. Ultrasound is used as an adjunct to enhance initial assessment of a patient, where image assessment and diagnosis occurs in real-time. The expert reviewers were blinded to any patient demographic, history or clinical assessment findings. They found assessing the images outside the dynamic clinical environment challenging. Conversely, the ARP’s did not describe such difficulties – as they interpreted images in real-time during clinical practice, they may have had the benefit of additional clinical information available at the scene.

It has been recognised that specific training in POCUS for non-physicians varies among health authorities, regulatory bodies, and employers [5]. The ARP’s in this study are advanced practitioners – all of whom had completed accredited training courses. Their advanced, specialised role may limit the study’s external validity, as they may not be representative of the wider non-physician population – or of those working purely in the in-hospital environment. In contrast, the study was conducted in the ‘real world environment’ against pragmatic challenges that are certain to be experienced by other clinicians, and in other services.

As part of the data analysis, a retrospective power calculation was undertaken. This calculation established that the number of images required to detect a statistically significant difference with the ARP’s being 86.4% accurate in their interpretation of image adequacy was 66 images. Therefore, the study was significantly underpowered, and a type-2 error cannot be excluded. A further, adequately powered, study should be undertaken. This could also be used to address other methodological challenges, for example the assumption of expert reviewer infallibility.

## Conclusions

This study demonstrated that Ultrasound of the lung can be used effectively by non-physicians to diagnose the presence or absence of pneumothorax in the pre-hospital environment without a statistically significant difference to expert review, allowing for the limitation of inadequate sample size. This can be achieved in austere environments; against the challenges of inclement weather, ambient light and the distracting nature of working in dynamic, high-risk clinical circumstances. Even considering the small number of patients involved, the correct diagnosis of pneumothorax - or absence thereof- has potentially significant implications for improving patient safety and reducing morbidity by informing decision making with regard to emergency surgical interventions in the pre-hospital arena.

## Data Availability

The datasets used and/or analysed during the current study are available from the corresponding author on reasonable request.

## Abbreviations

ARP: Advance Retrieval Practitioners
POCUS: Point of Care Ultrasound
CXR: Chest X-ray
EMRS: Emergency Medical Retrieval Service
ScotSTAR: Scottish Specialist Transfer and Retrieval
UK: United Kingdom
SLS: Sliding lung sign
HEMS: Helicopter Emergency Medical Service
EM: Emergency Medicine
RCEM: Royal College of Emergency Medicine
FICE: Focused Intensive Care Echo
CT: Computerised tomography
